# Editorial: Temporal bone histopathology: updates for neurotology

**DOI:** 10.3389/fneur.2025.1731926

**Published:** 2025-11-18

**Authors:** Bilen Korra Gelaye, Mohammad Moghimi, Miriam Redleaf

**Affiliations:** 1 Department of Otolaryngology/Head and Neck Surgery, University of Illinois at Chicago, Chicago, IL, United States; 2 Department of Biomedical Engineering, Comprehensive Cancer Center, Center for Artificial Intelligence Research, Center for Remote Health Monitoring, Translational Eye and Vision Research Center, Wake Forest University School of Medicine, Winston-Salem, NC, United States; 3 Otopathology Laboratory, University of Chicago, Chicago, IL, United States

**Keywords:** temporal bone, otopathology, neurotology, pathoneurology, otology

The field of temporal bone histopathology has long provided critical insights into disorders affecting hearing and balance. Harvested post-mortem, the temporal bones undergo a meticulous process of decalcification, embedding, sectioning, and staining—a cycle that spans approximately 12 months. This slow, detailed examination offers a window into otologic and vestibular diseases through specialized laboratories around the world, where decades of accumulated samples reveal the nuances of these complex conditions.

For more than a century, these serial temporal bone sections have been the bedrock of discovering and understanding the anatomic structures and relationships of the ear. Historically, any and all aspects of the human ear have been explored through temporal bone serial sections. These include the otic capsule and its contents, the middle ear and its contents, the eustachian tube, neural structures, vascular structures, aeration pathways, and the mastoid/petrous pyramid. Most of our fundamental understanding of normal function and disease has been supported by these visual slides. Despite the rich data that these numerous histopathologic studies have provided, there now exists a strange disregard toward the field of temporal bone histopathology. Therefore, one goal of this Research Topic was to revive some interest in temporal bone histopathology and to remind our community of the irreplaceable value of these collections and this field.

In addition, this Research Topic aimed to highlight the transformative nature of temporal bone histopathology. Contributors focused on showcasing the enduring value of such images. The studies in this Research Topic enhance the understanding and treatment of vestibulo-auditory disorders.

Included in this Research Topic are two reports on clinical findings. The first of these two articles is a classic case study that presents the clinical problem and otopathology of facial paralysis due to disease metastatic to the temporal bones. This article outlines the sites in the external auditory canal for metastatic malignancies (Garulo et al.). The second clarifies external canal cholesteatoma staging and reviews distribution of canal cholesteatomas as reported in the literature (He et al.).

The core of this Research Topic, however, speaks to the fundamentals of temporal bone histopathologic work. As mentioned above, traditional methods of temporal bone preparation require months of decalcification. A new method is reported here that uses microwaves and serial trimming of the specimens to almost halve the decalcification time and thus can accelerate investigations of targeted otologic diseases (Sagi et al.). Such an innovation in technique advances our field and can further reduce barriers to study of specific disease specimens.

Another contribution to this Research Topic meticulously outlines the structure, gene products, and gene loci for the components of a functioning tectorial membrane (Bian et al.). This article harkens back to more classic studies of fundamental otologic anatomy but with a new twist: this meticulous enumeration of loci and gene product histologic sites within the tectorial membrane explicitly links the tectorial membrane structures to hearing loss pathologies.

Finally, another contribution looks at changes within the cochlea when a neurofibromatosis type 2 vestibular schwannoma is present in the internal auditory canal (Al-Asad et al.). This rather ingenious study addresses the clinical puzzle of the deterioration of sensorineural hearing when the vestibular schwannoma has not been expanding. Immunohistochemical cell type markers (TUJ1, SOX10, and IBA1) were used to quantify neural, glial immune cells in the turns of the cochlea in normal and neurofibromatosis type 2-associated schwannoma. The authors' answer is to demonstrate changes in the cochlea when a schwannoma is present in the internal auditory canal. Such a study is classic temporal bone histopathology work.

In sum, the Research Topic redirects the community's attention toward the classic field of temporal bone histopathology. The content of these articles furthers our awareness of the clinical behavior of otologic disease. And the anatomic studies expand both our knowledge of the physical locations of gene products in the cochlea and link variations of those products to hearing loss. These classic collections of disease and normal specimens will continue to be the bedrock of otologic understanding for years to come.

